# Clonal Evolution and Therapeutic Resistance in Solid Tumors

**DOI:** 10.3389/fphar.2013.00002

**Published:** 2013-01-28

**Authors:** Michael T. Barrett, Elizabeth Lenkiewicz, Lisa Evers, Tara Holley, Christian Ruiz, Lukas Bubendorf, Aleksander Sekulic, Ramesh K. Ramanathan, Daniel D. Von Hoff

**Affiliations:** ^1^The Translational Genomics Research InstituteScottsdale, AZ, USA; ^2^Mayo Clinic ArizonaScottsdale, AZ, USA; ^3^Institute for Pathology, University of BaselBasel, Switzerland; ^4^Dermatology, Mayo Clinic ArizonaScottsdale, AZ, USA; ^5^Virginia Piper Cancer Center, Scottsdale HealthcareScottsdale, AZ, USA

**Keywords:** solid tumors, flow cytometry, clonal evolution, aCGH, next generation sequencing

## Abstract

Tumors frequently arise as a result of an acquired genomic instability and the subsequent evolution of neoplastic populations with variable genomes. A barrier to the study of the somatic genetics of human solid tumors *in vivo* is the presence of admixtures of non-neoplastic cells with normal genomes in patient samples. These can obscure the presence of somatic aberrations including mutations, homozygous deletions, and breakpoints in biopsies of interest. Furthermore, clinical samples frequently contain multiple neoplastic populations that cannot be distinguished by morphology. Consequently, it is difficult to determine whether mutations detected in a sample of interest are concurrent in a single clonal population or if they occur in distinct cell populations in the same sample. The advent of targeted therapies increases the selection for preexisting populations. However the asymmetric distribution of therapeutic targets in clonal populations provides a mechanism for the rapid evolution of resistant disease. Thus, there is a need to not only isolate tumor from normal cells, but to also enrich distinct populations of clonal neoplastic cells in order to apply genome technologies to identify clinically relevant genomic aberrations that drive disease in patients *in vivo*. To address this we have applied single and multiparameter DNA content based flow assays to the study of solid tumors. Our work has identified examples of clonal resistance to effective therapies. This includes androgen withdrawal in advanced prostate cancer. In addition we demonstrate examples of co-existing clonal populations with highly aberrant genomes and ploidies in a wide variety of solid tumors. We propose that clonal analysis of tumors, based on flow cytometry and high resolution genome analyses of purified neoplastic populations, provides a unique approach to the study of therapeutic responses and the evolution of resistance.

## Introduction

Nowell (1976) proposed that tumors arose as a result of an acquired genomic instability and the subsequent selection and evolution of clonal populations of neoplastic cells with unique patterns of aberrations. Consequently, each patient’s cancer may evolve and become dependent on distinct sets of selected aberrations during its clinical history. This clonal evolution model was based on studies primarily of liquid tumors using low resolution cytogenetics that showed uniform karyotypes of all cells in individual patient samples, biochemical assays, and allele-specific chromosome X expression of glucose-6-phosphate isoenzymes in all cells of a variety of neoplasms in heterozygous women, and immunology studies of homogeneous immuno-globulins in plasma cell tumors. Furthermore this model predicted that clonal behavior would mediate emergence of resistance even after an initial clinically significant response. Advances in genome technologies, notably oligonucleotide-based arrays and next generation sequencing (NGS) platforms, have greatly increased the resolution potential for studying the basis of therapeutic resistance in human cancers. A number of recent NGS studies have proposed evolutionary models of tumor development and progression based on heterogeneous patterns of mutations in primary and metastatic lesions (Gerlinger et al., 2012). These advances in technology and cancer genomics provide a rich opportunity to study the basis of therapeutic responses and the evolution of resistance.

A fundamental hypothesis in cancer biology is that the genes and cellular pathways deregulated by those selected events in each tumor genome represent enriched candidates for developing diagnostic markers and therapeutic targets. However, the cellular heterogeneity of clinical samples and the genetic diversity of cancer genomes are barriers to the translation of genomics for improved patient care. The presence of admixtures of non-neoplastic cells in patient samples can obscure the detection of somatic aberrations including mutations, homozygous deletions, and breakpoints in biopsies of interest. Furthermore, clinical samples frequently contain multiple neoplastic populations that cannot be distinguished by morphology based methods (Rabinovitch et al., 1999; Maley et al., 2006). Consequently, it is difficult to determine whether mutations and aberrations in a sample of interest are concurrent in a single tumor population or if they occur in distinct cell populations in the same sample. Thus, there is a need to not only isolate tumor from normal cells, but to also enrich distinct populations of clonal neoplastic cells in order to study cancer genomes in patients *in vivo*.

Cancer genome studies have relied primarily on two main approaches for selecting and preparing samples for analyses. The first is to pre select biopsies that exceed an arbitrary threshold for tumor cell content and necrosis based on histological methods such as H&E staining (Cancer Genome Atlas Research, 2011; Guichard et al., 2012; Nik-Zainal et al., 2012). However many samples fail the criteria; this is especially true for tumors arising in solid tissues where a high degree of tissue heterogeneity, with varied admixtures of reactive stroma, inflammatory cells, and necrosis in immediate contact with tumor cells, is a very common feature. Laser capture microdissection (LCM) can enrich tumor cell content prior to analysis. However LCM is limited in ability to objectively distinguish multiple clonal populations in single biopsies and in processing throughput for heavily admixed samples. The second approach is to passage biopsies either in tissue culture or in mouse xenografts (Jones et al., 2008). These methods apply a selective pressure on the complex mixtures of cells and clones present in a patient sample. Furthermore they can be time consuming, labor intensive, and cannot be efficiently applied in most clinical settings. Consequently the number of samples that can be successfully passaged varies from site to site, and the biological complexity and clinical context of the patient sample may not be reflected in the final processed sample. More recently, studies have used mixtures of tumor and normal cells to estimate thresholds for the detection of somatic mutations to compensate for the complex composition and cellular admixtures of clinical samples (Biankin et al., 2012). However these methods do not fully account for clonal heterogeneity, are dependent on depth of reads across each genome of interest, and require additional processing and filtering of increasingly complex data sets.

A major objective for cancer genome studies is to distinguish “driver” aberrations that target clinically significant signaling pathways from “passenger” aberrations that arise as a result of the biological background in genomically unstable tumors. A common approach for identifying clinically relevant cancer genome aberrations is to characterize lesions, including any loss or any gain for each chromosome, occurring at rates that are statistically significant in samples of interest (Aguirre et al., 2004; Weir et al., 2007; Chen et al., 2008; Kimmelman et al., 2008; Bredel et al., 2009; Liu et al., 2009). Single copy losses and gains occur as part of the random events associated with genomic instability present in tumor genomes. Thus many cancer genome studies determine a statistical threshold based on the background rates of losses and gains for detecting selected copy number changes. However this requires relatively large numbers of patient samples to account for the genomic instability and the patient specific variations typically present in cancer genomes. Furthermore recent NGS based studies have shown that the genomic landscapes of most solid tumors contain a wide variety of low prevalent mutations (McKenna et al., 2010; Biankin et al., 2012). Thus a fundamental challenge in the translation of cancer genomes for improved patient outcomes is the identification of selected genomic aberrations including somatic mutations that target clinically relevant signaling pathways in patients *in vivo*.

In order to address the complexity and heterogeneity of clinical samples we have applied highly quantitative DNA content based flow cytometry assays to identify and subsequently sort distinct fractions of tumor and non-tumor populations in each sample of interest (Ruiz et al., 2011; Liu et al., 2012). Flow cytometry cell sorters can select and objectively measure individual particles such as cells or nuclei using desired features defined by fluorescent and light scattering parameters in a flow stream. Once identified desired subpopulations can be deflected in the stream by an electric field and collected as pure objectively defined populations. Recent advances in this technology combine high efficiency flow rates with the detection of relatively rare events in dilute samples enabling the application of flow cytometry to clinical biopsies from clinical tissues (Ibrahim and van den Engh, 2003, 2008). For example, there are well established DNA staining based methods for isolating nuclei of aneuploid and diploid neoplastic populations from solid tumor samples (Rabinovitch, 1994; Glogovac et al., 1996). Tumor populations can be objectively and quantitatively purified to greater than 95% purity for molecular analyses even in heavily admixed clinical samples. In addition these methods can be combined with either proliferation markers such as Ki67 or tissue specific markers such as cytokeratins to purify distinct populations of diploid neoplastic cells from samples of interest (Prevo et al., 1999; Loo et al., 2004; Galipeau et al., 2007). Flow sorting has been successfully used for a variety of human neoplasias including breast, esophageal, lung, and colon carcinomas (Lofberg et al., 1992; Tanaka et al., 1995; Takanishi et al., 1996; Barletta et al., 1998; Loo et al., 2004; Lai et al., 2007). Recently we have developed methodologies that enable the study of flow sorted clinical samples with high definition whole genome assays, including oligonucleotide array based comparative genomic hybridization (aCGH) and NGS (Ruiz et al., 2011; Holley et al., 2012). The application of clonal analysis, based on whole exome and genome measurements of purified populations from clinical samples, provides a highly favorable approach to studying therapeutic responses and advancing more effective targeted therapies.

## Materials and Methods

Pancreatic ductal adenocarcinoma (PDA) samples were obtained under a WIRB protocol (20040832) for an NIH funded bio specimen repository (NCI P01 Grant CA109552) and two AACR/Stand up to Cancer (SU2C) sponsored clinical trials 20206-001 and 2026-003. Additional PDA samples as well as prostate carcinoma and ovarian carcinoma samples were obtained with approved consent of the Ethics Committee of Basel (252/08, 302/09). The melanoma tissue samples analyzed in this study were obtained under the Institutional Review Board approved protocols at Mayo Clinic. In all cases the tissues were acquired and the study was conducted according to Good Clinical Practice and the Declaration of Helsinki Principles with informed consent from each patient.

### Fresh tissue sample preparation and flow sorting

Wherever possible clinical samples are collected in liquid nitrogen and stored at −80°C. These include needle biopsies (e.g., 18 g), surgically resected tissues, pleural effusions, and core needle biopsies from various organ sites. Alternatively samples may be collected in small aliquots of tissue culture media supplemented with 10% DMSO and held on wet ice. These can then be transferred to −80°C for long term storage. The use of media with DMSO provides further tissue preservation in those cases where multiple sampling of the tissue involves cycles of freeze thawing. Prior to sorting each biopsy is quickly thawed on ice then minced in the presence of NST buffer and DAPI according to published protocols (Rabinovitch et al., 2001; Maley et al., 2006; Galipeau et al., 2007). Nuclei are mechanically disaggregated then filtered through a 40-μm mesh prior to flow sorting with an Influx cytometer (Becton-Dickinson, San Jose, CA, USA) with ultraviolet excitation and DAPI emission collected at >450 nm. DNA content and cell cycle are analyzed using the software program MultiCycle (Phoenix Flow Systems, San Diego, CA, USA).

### Formalin fixed paraffin embedded sample preparation and flow sorting

Formalin fixed paraffin embedded (FFPE) samples are fixed in formalin at the time of collection then stored according to routine pathology methods. Prior to sorting excess paraffin is removed with a scalpel from either side of 40–60 μm scrolls to reduce accumulation of debris during the sorting process. Each scroll is collected into individual microcentrifuge tubes then washed three times with 1 ml Xylene for 5 min to remove remaining paraffin. Each sample is rehydrated in sequential ethanol washes (100% 5 min ×2, then 95, 70, 50, and 30% ethanol) and washed two times in 1 ml of 1 mM EDTA pH 8.0. A 1-ml aliquot of 1 mM EDTA pH 8.0 is added to the samples and incubated at 95°C for 80 min to facilitate the removal of protein cross-links present in FFPE tissue. Samples are then cooled to room temperature for ≥5 min, followed by addition of 300 μl PBS pH 7.4 and gentle centrifugation for 2 min at 3.6 × *g*. The supernatant is carefully removed and the pellet washed three times with 1 ml PBS pH 7.4/0.5 mM CaCl_2_ to remove EDTA. Each sample is then digested overnight (6–17 h) in 1 ml of a freshly prepared enzymatic cocktail containing 50 units/ml of collagenase type 3, 80 units/ml of purified collagenase, and 100 units/ml of hyaluronidase in PBS pH 7.4/0.5 mM CaCl_2_ buffer. Each enzyme is rehydrated with PBS pH 7.4/0.5 mM CaCl_2_ buffer then stored at −20°C immediately prior to addition to the cocktail mixture. Following overnight digestion 500 μl NST is added to each sample to facilitate pelleting. Samples are then centrifuged for 5 min at 3000 × *g*, after which pellets are resuspended in 750 μl of NST/10% fetal bovine serum and then passed through a 25-G needle 10–20 times. The samples are filtered through a 35 μm mesh and collected into a 5 ml Polypropylene round bottom tube. The mesh is rinsed with an additional 750 μl of NST/10% fetal bovine serum and placed on ice while processing remaining samples. The total volume in the tube for each sample is approximately 1.5 ml. An equal volume of 20 μg/ml DAPI is then added to each tube to achieve a final concentration of 10 μg/ml DAPI prior to flow sorting with an Influx cytometer with ultraviolet excitation (Becton-Dickinson, San Jose, CA, USA). The optimal settings for sorting FFPE samples with the Influx sorter are as follows: drop formation is achieved with piezo amplitude of 6–10 V and a drop frequency of 30 kHz. The sort mode is set to purity yield with a drop delay of 31.5–32. Sheath fluid pressure is typically 17–18 psi with a 100 μm nozzle. For single parameter DNA content assays DAPI emission is collected at >450 nm. In each sorting experiment we used one or more 50 μm FFPE scrolls to obtain sufficient numbers of intact tumor nuclei for subsequent molecular assays. DNA content and cell cycle are then analyzed using the software program MultiCycle (Phoenix Flow Systems, San Diego, CA, USA).

Gating based on DNA content provides a robust quantitative measure for identifying and sorting tumor populations from samples of interest. The ploidy and the relative distribution of each population present in a biopsy can be recovered by fitting the G_0_/G_1_ and G_2_/M peaks as Gaussian curves and the S phase distribution as a Gaussian broadening distribution. The DNA content histograms from tumor tissue are frequently suboptimal (broad c.v’s, high debris and aggregation) and often complex (multiple overlapping peaks and cell cycles) with frequent skewing and non-Gaussian peak shapes. This is even truer for FFPE specimens that often contain higher levels of damaged or fragmented nuclei (debris) resulting in events usually most visible to the left of the diploid G_1_ peak and that fall rapidly to baseline. For reproducible phase measurements we typically acquire 10,000 events. However if a substantial proportion of events are from debris or aggregates, the total number of events acquired must be correspondingly higher in order to assure the required minimum number of intact single nuclei for accurate curve fitting. Despite these challenges distinct populations can be efficiently sorted from routinely prepared FFPE samples.

### DNA extraction

The genomic DNA from sorted nuclei is extracted using an amended protocol from QIAamp^®^ DNA Micro Kit from Qiagen (Valencia, CA, USA). Briefly each sorted sample is resuspended in 180 μl buffer ATL and 20 μl proteinase K then incubated for 3 h at 56°C for complete lysis. Samples are bound and washed according to QIAamp^®^ DNA Micro Kit instructions, eluted into 50 μl of H_2_0, then precipitated overnight with 5 μl 3 M sodium acetate and 180 μl 100% EtOH. Each sample is then centrifuged for 30 min at 20,000 × *g*, washed in 1 ml of 70% EtOH for 30 min at 20,000 × *g*. The samples are carefully decanted and the DNA pellet was dried by speed vacuum then resuspended in a small volume (e.g., 10–50 μl) of H_2_O for final concentrations suitable for accurate quantification.

### DNA amplification

To make efficient use of sorted clinical samples we have optimized whole genome amplification methods to generate DNA templates suitable for whole genome and exome analyses. Sorted populations from fresh frozen samples can be amplified with highly processive enzymes such as phi29 that require intact genomic DNA as starting material. Extracted fresh frozen sourced genomic DNA is amplified using the Illustra GenomiPhi V2 Amplification kit from GE Healthcare Bio-sciences, Corp. (Piscataway, NJ, USA) according to our published protocols (Ruiz et al., 2011). Typically we use >50 ng genomic DNA from each sorted population as input to ensure linear amplification across the genome.

For FFPE samples the DNA templates are not suitable for amplification protocols with highly processive polymerases. Thus we validated an alternative method based on single primer based amplification of size selected double strand DNA input (Holley et al., 2012). Genomic DNAs from sorted FFPE samples are amplified using Ovation^®^ WGA FFPE System from NuGEN^®^ Technologies (San Carlos, CA, USA). DNA is processed in accordance with Ovation^®^ WGA FFPE standard SPIA protocol with an alternate T7 endonuclease fragmentation step. These protocols require input from 50,000 sorted nuclei as starting material to ensure a linear amplification. Resulting amplified products are either used as template for aCGH analysis or processed with the Nugen Encore ds-DNA module according to the supplier’s instructions in order to generate double-stranded (ds) end-repaired DNA as input for library suitable for NGS. In all cases A 50–100 ng aliquot of pooled 46,XX DNA (Promega, Madison, WI, USA) is amplified with the matching amplification protocol to generate a suitable reference for each aCGH experiment using amplified DNA templates. The quality of each amplification product is assessed by gel electrophoresis prior to downstream analysis.

### aCGH analysis

Fresh frozen phi29 amplified and FFPE non-amplified DNAs are treated with DNAse 1 prior to Klenow-based labeling. High molecular weight phi29 templates are digested for 30 min while the smaller fragmented FFPE samples are digested for only 1 min. In each case 1 μl of 10× DNase 1 reaction buffer and 2 μl of DNase 1 dilution buffer are added to 7 μl of DNA sample and incubated at room temperature then transferred to 70°C for 30 min to deactivate DNase 1. In contrast the amplified FFPE sourced DNAs do not require DNase 1 treatment prior to Klenow-based labeling. In all cases sample and reference templates are labeled with Cy-5 dUTP and Cy-3 dUTP respectively using a BioPrime labeling kit (Invitrogen, Carlsbad, CA, USA) according to our published protocols (Ruiz et al., 2011). All labeling reactions are assessed using a Nanodrop assay (Nanodrop, Wilmington, DE, USA) prior to mixing and hybridization to 400 k CGH arrays (Agilent Technologies, Santa Clara, CA, USA) for 40 h in a rotating 65°C oven. All microarray slides are scanned using an Agilent 2565C DNA scanner and the images are analyzed with Agilent Feature Extraction version 10.7 using default settings. The aCGH data are assessed with a series of QC metrics then analyzed using an aberration detection algorithm (ADM2; Lipson et al., 2006). The latter identifies all aberrant intervals in a given sample with consistently high or low log ratios based on the statistical score derived from the average normalized log ratios of all probes in the genomic interval multiplied by the square root of the number of these probes. This score represents the deviation of the average of the normalized log ratios from its expected value of zero and is proportional to the height *h* (absolute average log ratio) of the genomic interval, and to the square root of the number of probes in the interval.

### Exome library preparation

A total of 3 μg of high-quality DNA template with a 260/280 ratio between 1.8 and 2.1 is fragmented to a target size of 150–200 base pairs on the Covaris E210 system. Fragmentation is verified on a 2% TAE gel and fragmented samples are end-repaired using New England Biolab’s NEB Next kit (Ipswich, MA, USA). Repaired samples are adenylated at the 3′ end using the NEBNext kit, and Illumina indexed adapters are next ligated onto A-tailed products. Samples are next PCR amplified using Herculase II polymerase and purified. Samples are then run on an Agilent Bioanalyzer to verify amplification and to quantify samples. Samples are adjusted to 147 ng/μL for 24 h hybridization to exonic RNA probes using Agilent’s SureSelect All Exon 50 Mb Plus kit, which contains 561,823 probes targeting 202,124 exons. Captured products are next selected for, purified, and PCR amplified. Final libraries are verified and quantified using an Agilent Bioanalyzer.

### Paired end next generation sequencing

Libraries are denatured using 2 N NaOH and diluted with HT2 buffer (Illumina). One percent of denatured and diluted phiX is spiked into each lane to allow for error rate reporting on the HiSeq. Cluster generation is performed using Illumina’s cBot and HiSeq Paired End Cluster Generation Kit. Flow cells are paired end sequenced on Illumina’s HiSeq 2000 using Illumina’s HiSeq Sequencing Kit. Raw sequencing data are converted to standard FASTQ format using CASAVA pipeline with in-house custom scripts^1^^,^^2^. FASTQC program is used for quality control and all reads are trimmed to 90 high-quality base pairs. In order to generate at least 100 million pass filter reads for each exome library, two lanes of a HiSeq 2000 flowcell are sequenced for each of the FFPE and fresh frozen exomes. Data is aligned to hg18 assembly of human genome using BWA sequence alignment software (version 0.5.9) and raw alignment BAM files are further processed for quality recalibration, duplicate removal, and local realignment using a custom in-house pipeline based on Picard and GATK tools^3^ (Li and Durbin, 2009; McKenna et al., 2010; DePristo et al., 2011). For each sample, variants are called from BAM files using samtools and varscan using a minimum coverage cut-off of 10, and only those variants that are called by both algorithms are retained (Koboldt et al., 2009; Li et al., 2009).

## Results

### Clonal analysis: Single parameter DNA content based flow sorting

**Figure 1:**
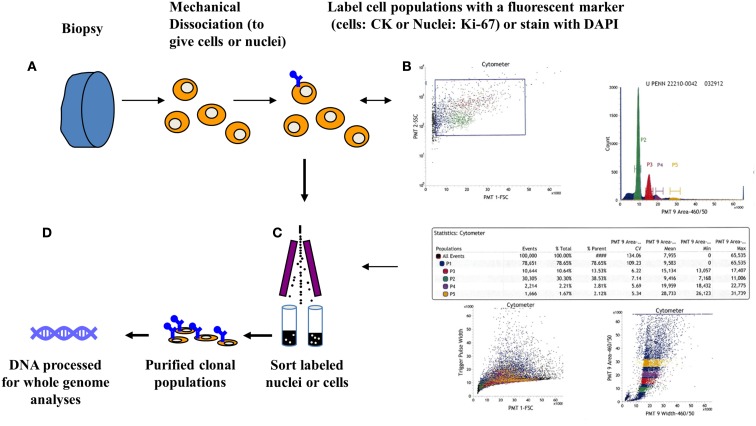
**Clonal profiling: flow sorting neoplastic cells from solid tissue biopsies**. **(A)** Biopsies are minced in the presence of DAPI then mechanically disaggregated to create single nuclei suspensions. These are then flow sorted in single or multiparameter assays. **(B)** Scatter plot, displaying events during sorting, are used to generate histograms to identify then subsequently sort populations of interest. Table displays quantitative analysis of the four peaks (P1–P4) detected in a pancreatic adenocarcinoma sample. **(C)** Each peak was sorted and collected into individual tubes. **(D)** Genomic DNA from each purified population is extracted then processed for assays of interest including whole genome and whole exome analyses.

Flow cytometry methods can discriminate distinct populations in each biopsy of interest based on one or more features. The basic principle for flow sorting is to interrogate single particles in suspension for desired parameters. These include ploidy as defined by the mean DNA content, as well as differentiation or cell lineage markers. Clonal populations in tumors can then be distinguished by differences in ploidy, copy number aberrations, mutations, or differentiation. For solid tumor samples tissue are mechanically disaggregated and the nuclei suspended in the presence of DAPI. The isolation of nuclei provides an efficient mechanism to prepare single particle suspensions that can be interrogated in a flow stream. A hallmark of many human cancers is the development of genomic instability and the evolution of aneuploid tumor cell lineages. Thus for many solid tumors DNA content assays can be very effective in identifying and subsequently sorting neoplastic populations for genomic analysis. For example we identified four distinct populations in a biopsy from a PDA surgical resection (Figure 1). Each of these populations was collected in our sorting assay then processed for whole genome analysis. These included a genomically normal diploid population as well as a small tumor aneuploid population. The ability to collect four simultaneous streams in these assays optimizes the use of each clinical sample and provides isogenic populations for analysis.

### Tumor markers and therapeutic targets

Pancreatic ductal adenocarcinoma is a highly lethal tumor type that is difficult to molecularly characterize at the biopsy level due to complex genomes and heterogeneous cellularity, as cancer cells represent on average only 25% of the cells within the tumor (Seymour et al., 1994). Previous studies of PDA have relied on tumor derived cell lines and xenografts to obtain samples for genome studies (Jones et al., 2008). However these methods create selective pressures on tumor cells, are typically labor intensive, and require fresh tissues limiting their utility for patient based studies. More recently, studies have used mixtures of tumor and normal cells to estimate thresholds for the detection of somatic mutations to compensate for the complex composition and cellular admixtures of clinical PDA samples (Biankin et al., 2012). In contrast we apply our flow sorting methods to clinical samples and profile the genomes of each sorted population. These highly purified clonal preparations are used for high definition genomic analyses to determine the landscape of aberrations present in each tumor. The ability to profile patient samples regardless of tumor cell content provides an unbiased approach to the study of PDA genomes.

**Figure 2:**
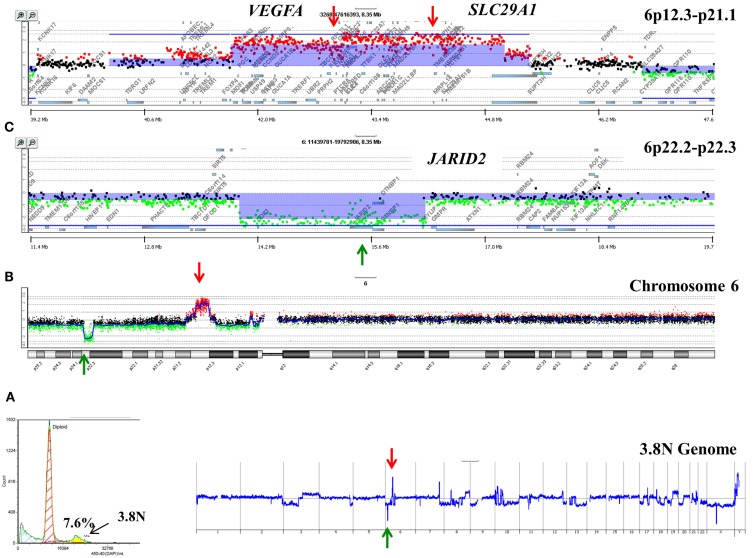
**Clonal analyses of pancreatic ductal adenocarcinoma (PDA) biopsy P1026Z**. **(A)** DAPI-based DNA content analysis detected a 3.7N population representing 7.6% of the cellular content of the biopsy. Only the 3.7N population showed homozygous deletions and focal amplicons. **(B)** Chromosome 6 and **(C)** locus-specific views of the *JARID2A* homozygous deletion and the *VEGFA SLC29A1* amplicon in the 3.7N PDA genome. Blue shade areas denote ADM2 aberrant intervals.

A striking observation in our ongoing studies of PDA is the unique nature of the genomes profiled from each patient. For example a 3.7N aneuploid population representing <8.0% of a PDA sample had a focal amplicon on 6p21 as the most significant copy number gain in its genome (Figure 2). This high level focal amplicon includes the *VEGFA* gene and the nucleoside transporter gene *SLC29A* (ENT1) that regulates intracellular gemcitabine levels. The presence of these co-amplified genes suggests a clinical hypothesis that this tumor has a context of vulnerability to therapy based on anti-angiogenic agents and would be responsive to gemcitabine. In addition this same genome had a focal homozygous deletion targeting *JARID2*, a regulator of histone methyltransferase complexes during normal development. A current hypothesis in our PDA clinical studies is that loss of normal epigenetic regulation may make tumors responsive to combination treatments that include hypomethylation agents such as 5-azacytidine.

**Figure 3:**
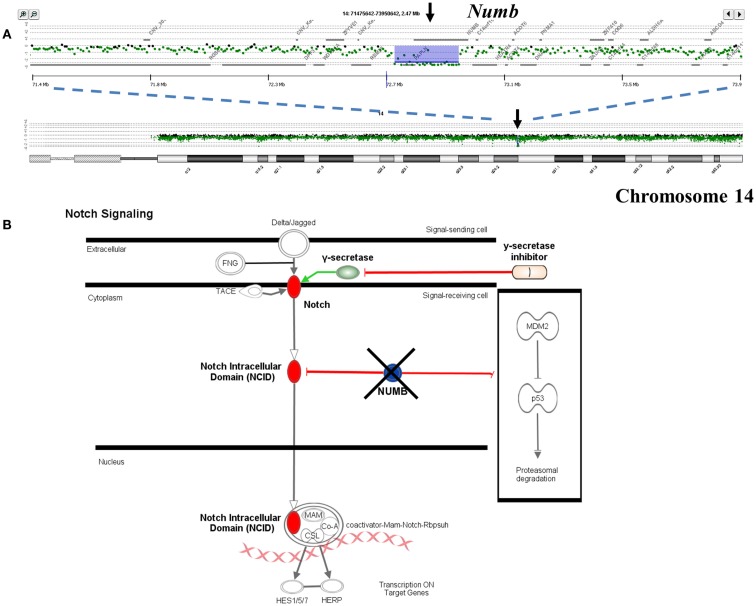
**Clinical significance of *NUMB* homozygous deletion in pancreatic ductal adenocarcinoma (PDA) genome**. **(A)** Homozygous deletion of the *Numb* tumor suppressor gene in an aneuploid clone isolated from a PDA sample. Shaded areas represent copy number aberrant regions called by ADM2 algorithm. **(B)** Concept map of *Numb* and its interactions with *Notch* and *TP53* pathways.

Single copy gains and losses can arise at high rates as a result of the genomic instability that is a hallmark of many tumors. In contrast, events such as homozygous deletions and focal high level amplifications typically require multiple independent genomic events and thus can represent biologically selected events in cancer genomes that target known and putative tumor suppressor genes and activated oncogenes. The identification of genes targeted by these selected events provides insights into signaling pathways that may drive the clinical behavior of individual tumors. For example previous reports have identified somatic mutations of *NUMB* in breast carcinoma and of *PARK2* in GBM, colon, and lung cancers (Colaluca et al., 2008; Veeriah et al., 2010). We have identified homozygous deletions in both of these genes providing evidence that their tumor suppressor function extends to PDA. The potential clinical significance of homozygous deletions is highlighted by the prediction that complete loss of *NUMB* results in increased Notch signaling which can be targeted by gamma secretase inhibition or by blocking the Notch receptor directly (Figure 3). These same genomic analyses have also identified homozygous deletions in a series of other known (e.g., *SMAD2*, *SMAD3*, *JARID2*) and putative (*SMYD3*, *USP25*) tumor suppressor genes that are selectively disrupted in PDA genomes. Given that many tumor suppressor genes targeted by homozygous deletion have frequent sequence mutations the latter provide highly favorable candidates for resequencing and IHC based assays.

The detection of the complete loss of genomic sequence is highly sensitive to as little as 5% admixtures of non-tumor cells in clinical samples (Zhao et al., 2004). This is consistent with recurring observations that fewer aberrations can be detected in patient samples *in vivo* than in passaged model systems (Jones et al., 2008; Leary et al., 2008; Parsons et al., 2008). However a limitation in many cancer genome studies is the inability to objectively discriminate single copy losses from homozygous deletions and to accurately map and determine high level amplification in cancer genomes arising in individual patient samples. Thus copy number data is typically reduced to reporting frequencies of any loss or any gain for each chromosome in relatively large cohorts of samples. This further limits the translational potential of the genomic study of cancers including those with variable complex genomes, rare cancers, and biopsies with high admixtures of genomically normal cells and insufficient tumor cell content (Aguirre et al., 2004). Furthermore, histology-based methods cannot readily distinguish whether aberrations in a tumor are present in a single cancer genome or if they are distributed in multiple clonal populations. Consequently current approaches for the analyses of cancer genomes are limited in their ability to determine the clinical context of each patient’s tumor.

**Figure 4:**
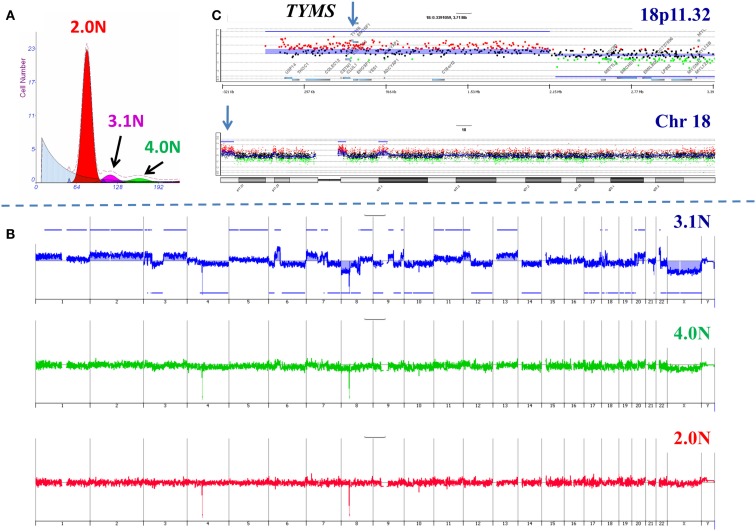
**Clonal analysis of pancreatic ductal adenocarcinoma (PDA) liver metastasis from SU2C clinical trial**. **(A)** DNA content flow sorting identified and purified three distinct peaks in the single biopsy. **(B)** The 2.0N and 4.0N populations were genomically normal while the 3.1N population had multiple copy number aberrations throughout its genome. **(C)** Focal amplicon on 18p11.32 that included the thymidylate synthase (*TYMS*) locus. Blue shade areas denote ADM2 aberrant intervals.

In order to further validate our clonal methods we have applied those to our ongoing Stand Up to Cancer (SU2C) PDA clinical trials. The first of these trials, 2026-001, involves molecular profiling of patients with previously treated metastatic disease. For each of the 35 patients in this trial one to three 18g needle biopsies from a liver metastasis was available for analysis. These samples were subsequently analyzed for a panel of IHC markers under CLIA conditions to identify a clinically actionable target. As a correlative samples of these same tissues were also processed for clonal analysis using single parameter DNA content sorting and aCGH for each sorted population. An example of these studies is a patient who had previously been treated with 5-FU based therapy (Figure 4). Three distinct populations were identified and subsequently sorted from the liver metastasis biopsy. The genome of each sorted population was then profiled by aCGH. The diploid and tetraploid fractions were genomically normal by copy number analysis. In contrast we detected a series of somatic genomic aberrations in the 3.1N population. These included a focal gain at 18p11.32 that included the thymidylate synthase (*TYMS*) locus whose protein product is targeted by 5-FU based therapies. IHC revealed an increased level of *TYMS*. This suggests that prior treatment resulted in amplification of this region and increased protein levels and gave rise to the therapeutic resistance that arose in this patient during treatment.

### Clonal heterogeneity in single biopsies

**Figure 5:**
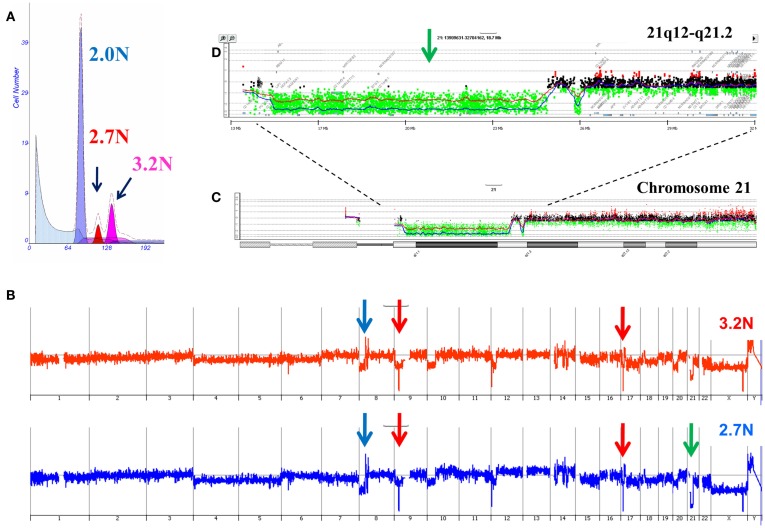
**Clonal analysis of pancreatic ductal adenocarcinoma (PDA) liver metastasis from SU2C clinical trial**. **(A)** DNA content flow sorting identified and purified a diploid and two distinct aneuploid (2.7N, 3.2N) peaks in the single biopsy. **(B)** Whole genome copy number profiles of the 2.7N and 3.2N populations. **(C)** Aberration detection on chromosome 21 and **(D)** 21q12-q21.2 in both aneuploid populations. Blue shaded areas denote ADM2 aberrant regions.

Our ongoing clinical studies have provided a unique view of clonal heterogeneity and show that clonal evolution can be driven by different biological events in patient samples. For example the presence of distinct aneuploid populations with overlapping copy number aberration profiles suggests a mitotic event contributed to the heterogeneity of the tumor. In some of these cases simultaneous ploidies acquire relatively subtle copy number differences in their genomes. Single parameter DNA content sorting identified two distinct ploidies (3.2N and 2.7N) in a liver metastasis that shared a series of genomic aberrations including focal amplification of *FZD3* (8p21) and homozygous deletions of *MAP2K4* (17p12) and *CDKN2A* (9p21.3; Figure 5). In addition each of the clonal populations had a deletion with matching boundaries on 21q. In the 3.2N genome the deletion was homozygous as measured by a log_2_ratio value of <−3.0 for CGH probes in the interval. In contrast the same interval was only partially deleted in the 2.7N population. Given that the *MAP2K4* and *CDKN2A* deletions were homozygous in both populations these data suggests that the distinct aneuploid populations evolved after metastasizing to the liver and that the 3.2N clone acquired complete loss of this region as a result of ongoing genomic instability. The presence of these two distinct but highly related clonal populations in a single liver metastasis highlights the ongoing nature of evolution in PDA.

We have made similar observations of ongoing instability and evolution in a wide variety of solid tumors including melanoma, adrenal cortical carcinoma, breast cancer, hepatocellular carcinomas, and colon carcinoma. The significance of clonally diverging events and their possible role in resistance to therapy and disease progression is being followed in ongoing clinical trials. A fundamental hypothesis is that distinct clones will have differential responses to selective therapies. A challenge for clinical trials and advancing personalized therapies for patients with solid tumors will be to objectively measure genomic lesions in one or more genomes present in clinical samples of interest.

### Clonal heterogeneity in metachronous samples

**Figure 6:**
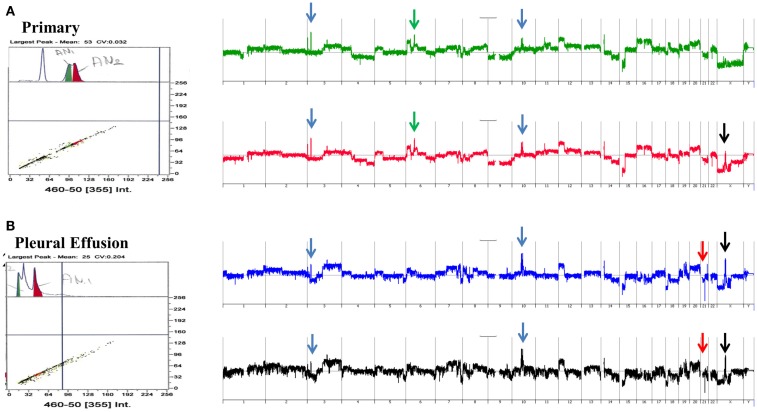
**Clonal analysis of matching metachronous primary ovarian tumor and pleural effusion**. **(A)** DNA content detection of two distinct aneuploid populations in primary tumor samples from 2009. Whole genome copy number plots for each sorted population. **(B)** DNA content detection of two distinct aneuploid populations in pleural effusion tumor samples from 2010. Whole genome copy number plots for each sorted population. Blue arrows shared aberrations; red arrows pleural effusion specific aberrations; green arrows primary specific aberrations; black arrows single primary and shared pleural effusion aberrations.

**Figure 7:**
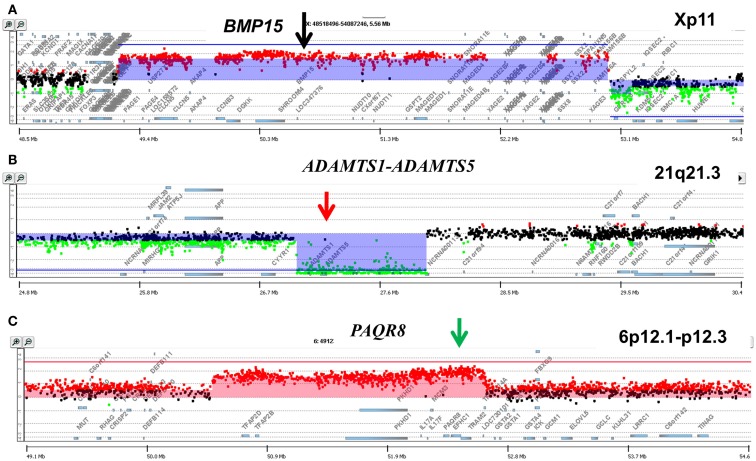
**Genomic aberrations and clonal analysis of matching metachronous primary ovarian tumor and pleural effusion**. **(A)** Chromosome X11p focal amplicon and *BMP15*. **(B)** Chromosome 21q21.3 homozygous deletion targeting *ADAMTS1-ADAMTS5*. **(C)** Chromosome 6p12.1-p12.3 amplicon and *PAQR8*. Blue shaded areas denote ADM2 defined aberrant intervals.

A striking example of clonal heterogeneity is shown in an ovarian carcinoma with biopsies from the primary tissue and a pleural effusion sample acquired 1 year later. DNA content sorting identified two aneuploid populations in each of the clinical samples (Figure 6). Strikingly the ploidy of each of the four populations was distinct. Two hypertetraploid populations were identified in the primary tumor while the pleural effusion sample had a hypodiploid and a hyperdiploid population. Each of these four aneuploid populations was sorted and profiled for copy number aberrations. The genomes of these four distinct populations had shared as well as distinct aberrations that further define the clonal heterogeneity of this tumor. For example the shared aberrations included high level focal amplicons on chromosomes 3p and 10q. We also detected aberrations that were present in one of the primary populations and in both of the populations detected in the pleural effusion. This included an amplicon on chromosome Xp11 that includes the *BMP15* locus (Figure 7A). The protein encoded by this gene is a member of the bone morphogenetic protein family which is part of the transforming growth factor-beta superfamily. This protein is believed to be involved in oocyte maturation and follicular development as a homodimer and by forming heterodimers with a related protein; Gdf9. In addition we detected a homozygous deletion at 21q that simultaneously deleted the metallopeptidases *ADAMTS1* and *ADAMTS5* that was present only in the pleural effusion populations (Figure 7B). The presence and patterns of these amplicons and homozygous deletions in these samples suggest that *BMP15* amplification contributed to clonal selection during progression, and that homozygous deletion of the metallopeptidases contributed to the establishment of metastatic lesions in this patient’s tumor.

However there were also aberrations that were present in the primary populations but absent in the pleural effusion samples. Specifically another focal amplicon on chromosome 6p targeting the progestin and adipoQ receptor family member VIII gene locus (*PAQR8*; Figure 7C). The latter encodes a steroid membrane receptor that binds progesterone and has been implicated in normal oocyte maturation. The absence of this amplicon in the pleural effusion samples suggests that the evolution of this tumor may be driven by progenitor populations lacking the 6p amplicon in a non-linear lineage. This relatively simple example using single parameter sorting, aCGH, and two chronological samples from a single patient highlight the potential complexity of clonal evolution during tumor progression.

### Clonal heterogeneity, evolution, and acquired resistance to targeted therapy

**Figure 8:**
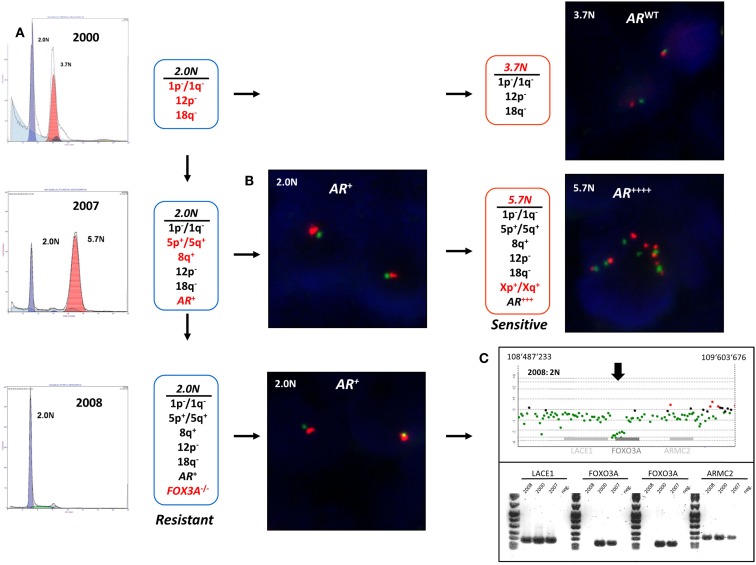
**Clonal analyses and evolution of androgen-independent prostate carcinoma**. Multiple populations were detected by DNA content flow cytometry over an 8-year time span during the clinical history of a prostate patient. Each population showed a series of shared (black) and novel (red) genomic aberrations. **(A)** DNA content histograms and CGH summaries for each biopsy. Diploid (2.0N) and aneuploid populations arose in 2000 and evolved during clinical history of disease. **(B)** FISH analysis of chromosome X (green centromere; red AR locus) and validation of AR amplicons arising in 2006. **(C)** PCR validation of homozygous deletion of FOXO3A gene in 2.0N population that evolved after AR withdrawal therapy in 2008.

The detection and sorting of more than one tumor population in a biopsy and the availability of multiple biopsies from individual patients extends the study of clinical phenotypes and the behaviors of clonal populations *in vivo*. For example the aneuploid populations with adverse histological features and multiple selected genomic aberrations including a 5.7N population with high level *AR* amplification that arose during the clinical history of a patient who developed advanced PC, were uniquely sensitive to therapeutic regimen and were erased after hormone withdrawal (Figure 8). The patterns of acquired clonal aberrations, including losses on chromosomes 1p, 1q, 12p, and 18q that persisted throughout progression, suggest that the aneuploid populations arose from diploid progenitors during the evolution of disease. Strikingly, the co-occurring diploid cells acquired a low-level focal *AR* amplicon after bilateral orchiectomy leading to increased sensitivity to remaining levels of adrenal testosterone, followed by homozygous deletion of *FOXO3A* in response to androgen blockage, resulting in the evolution of androgen-independent metastatic disease. The clonal relationships of these populations were further probed by resequencing a panel of 400 cancer related genes, including the AR gene, in the diploid and aneuploid populations from 2007 and 2008 biopsies. The AR gene was wild type in all three populations and there were no somatic mutations that further distinguished one population from the other. Thus our clonal analysis highlights the role of distinct but related tumor populations during the evolution of resistant disease. The distinct *AR* amplicons present in the diploid and the aneuploid populations that arose during the evolution of androgen-independent metastatic PC, and the homozygous deletion of the pro apoptotic *FOXO3A* gene would have been obscured in a conventional histologically prepared sample. We propose that similar clonal behaviors of tumors, involving target(s) asymmetrically distributed within a clonal lineage and the selection for pro survival genomic aberrations underlie clinical responses to existing and emerging targeted therapies. The fundamental hypothesis is that clonal selection in the presence of these agents will lead to rapid evolution and emergence of resistant tumors. We are currently testing these hypotheses in a number of tumor types.

### Clonal analysis: Multi parameter based flow sorting

**Figure 9:**
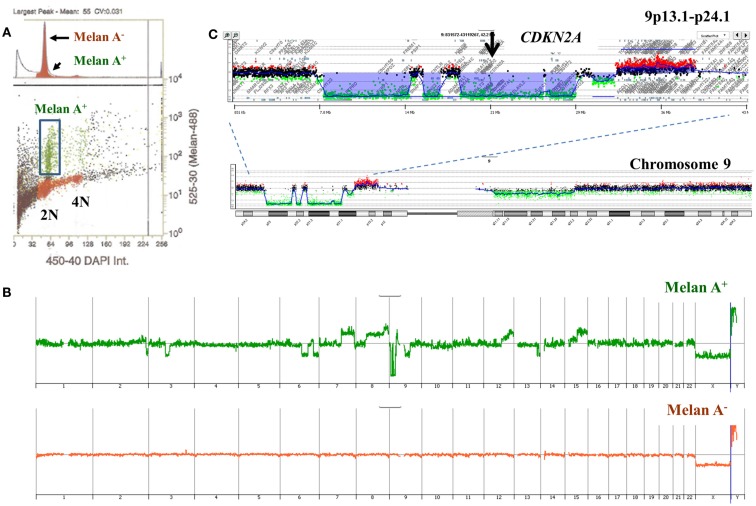
**Multiparemeter sorting and CGH analysis of melanoma lymph node biopsy**. **(A)** DNA content (*x*-axis) and melan A (*y*-axis) based sorting identified a small melan A+ diploid fraction. **(B)** Whole genome view of melan A− and melan A+ diploid genomes. **(C)** Chromosome 9 and 9p13.1-p24.1 views of homozygous deletion in diploid melan A+ genome. Blue shaded area denotes ADM2 defined aberrant interval.

A limitation of single parameter DNA content sorting strategies is the inability to resolve tumors that have a mean DNA content that cannot be distinguished from normal diploid cells by flow cytometry. One approach is to focus of the 4N peaks that may be present in the population. Studies in Barrett’s esophagus, a known risk factor for esophageal adenocarcinoma, have shown that 4N fractions greater than 6% arise in neoplastic tissue and are associated with known genomic events such as *TP53* mutations (Galipeau et al., 1996; Rabinovitch et al., 2001; Barrett et al., 2003). These elevated 4N populations are frequently enriched with the G_2_/M fractions of proliferating diploid cells as well as the G_0_/G_1_ fractions of tetraploid populations. Alternatively DNA content can be combined with a variety of markers to isolate populations based on tissue type, differentiation, or proliferation status. For example melan A combined with DNA content identified a diploid melanocyte population in a lymph node metastasis sample whose genome contained a series of aberrations including homozygous deletion at 9p21.3 (Figure 9).

**Figure 10:**
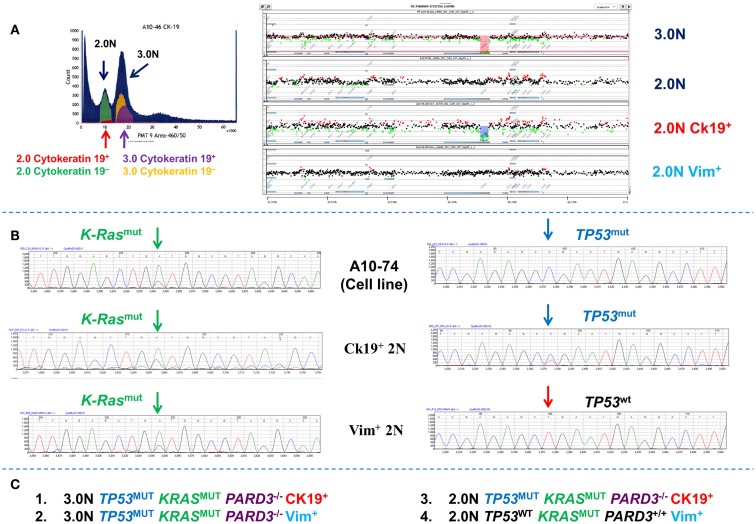
**Clonal analysis of diploid and aneuploid populations in a pancreatic ductal adenocarcinoma (PDA) biopsy**. **(A)** DNA content cytokeratin 19 multiparameter sort of PDA biopsy. A small (<5%) CK19+ fraction was detected and sorted from the biopsy. These were compared to total 2.0N, 3.0N, and vimentin+ 2.0N populations sorted from the same tissue. **(B)** Targeted resequencing of K-ras and TP53 in sorted CK19+ diploid, sorted Vim+ diploid, and patient derived cell line. **(C)** Summary of clonal content of PDA biopsy.

In addition to diploid tumor populations multiparameter sorting resolves clonal heterogeneity in individual samples that would otherwise be obscured by analysis of bulk samples. For example whole exome analysis of single parameter DNA content sorted 2.0N and 3.0N populations from a PDA biopsy identified a series of mutations and copy number aberrations in the aneuploid population (Figure 10). The total diploid sorted fraction from the PDA tissues was non-aberrant by aCGH analysis. However a low (<1–5%) number of reads for some mutations present in the aneuploid fraction (e.g., *KRAS*) were observed in the NGS data for the total diploid fraction. The total diploid peaks in DNA content based flow sorted tumor samples may contain admixtures of neoplastic and non-neoplastic cell types. To determine whether these low frequency mutation reads are present in distinct subpopulations of neoplastic cells we used a DAPI/cytokeratin 19 and a DAPI/vimentin flow assay to resort the biopsy into four distinct populations (Figure 10). The cytokeratin 19^+^ (CK19^+^) and the vimentin^+^ (Vim^+^) diploid populations each had the heterozygous *KRAS* mutation detected in the aneuploid population. However, only the small (1–5%) CK 19^+^ diploid population had the clonal homozygous *TP53* mutation and an aCGH profile, including a homozygous deletion of *PARD3*, which matched the 3.0N population. Thus the 2N CK 19^+^ population represents a co-existing diploid epithelial tumor population. In contrast the Vim^+^ diploid *KRAS*^mut^, *TP53*^wt^ population was normal by aCGH and represents a third clonal population in this biopsy that is either from an earlier stage of disease or is a non-progressing neoplastic population. Our ability to resort this tissue provides a unique approach to validate NGS results and confirm the presence of distinct clonal populations. We propose that this iterative approach can be used to exploit the detection of low frequency reads in NGS data of clinical samples and to provide even deeper clonal analysis.

## Discussion and Conclusion

DNA content based flow sorting of clinical samples provides highly purified populations of tumor cells for genomic analyses. Consequently the clonal behaviors of samples of interest, including the presence of distinct populations of tumor cells and their differential responses to therapies, can be studied in patients *in vivo*. We and others have shown that both tissue and clonal heterogeneity can vary widely even in adjacent biopsies from the same tumor (Ruiz et al., 2011; Gerlinger et al., 2012). Gating based on DNA content provides a highly reproducible quantitative measure for identifying and sorting tumor populations from samples of interest. For example a 3.0N population sorted from a FF PDA sample was detected 3 years later in an FFPE sample from the same tissue (Holley et al., 2012). Genomic analysis including whole exome sequencing, confirmed that the sorted 3.0N populations were identical. The sorting efficiencies of clinical samples can be significantly affected by the presence of debris, aggregates, and sliced nuclei. To maintain sorting efficiencies at relatively high levels (>80%) and high yields and purities of sorted samples the differential pressure of the core and the sheath fluids can be increased but cannot be ≥1. Slow sort rates while maintaining optimal differential pressure of flow stream improves efficiency of sorts and the overall yield of intact nuclei. However the greatest variable in our sorting was the origin of the tissue. For example triple negative breast cancer (TNBC) samples typically sort more efficiently than PDA samples for both fresh frozen and FFPE samples.

Our flow sorting based clonal genomic methods have been designed to leverage highly valuable clinical samples. These methods aim to overcome some of the most common limitations to the study of clinical samples including high admixtures of non-neoplastic tissue, small sample size, and the presence of high levels of necrosis. This extends the number of clinical samples that can be interrogated allowing a more unbiased survey of each tumor type. The most highly favorable samples for studying the clonal behaviors of tumors and their role in acquired resistance are clinical biopsies obtained during clinical trials. The challenge for genomic based investigations is to optimize the use of these increasingly valuable samples and to profile each of them in an unbiased and quantitative manner. Our ability to use FFPE tissues expands our clonal analysis to this vast resource of clinically annotated samples with patient follow-up data (Holley et al., 2012).

A fundamental hypothesis of our current studies is that the presence and behaviors of distinct diploid and aneuploid populations in clinical samples whose genomes contain both shared and unique will affect therapeutic responses and clinical outcomes. This will be of increasing significance with the ongoing development of more targeted therapies for human cancers. Current NGS based studies use increasingly deep coverage and novel algorithms to infer the presence of clonal populations in clinical samples (Cancer Genome Atlas Research, 2011; Nik-Zainal et al., 2012). In contrast our approach focuses on the identification and purification of distinct populations of interest prior to genomic analysis. Once identified these pure populations provide a clonal framework to map the mutational and copy number landscape of each tumors genome(s).

Previous studies with sorted clinical samples have highlighted the utility of purified sorted samples for epigenetic studies of loci of interest (Wong et al., 1997). Currently we are using multiparameter sorting strategies in combination with high resolution NGS based methylation assays to investigate the epithelial and mesenchymal components of tumors. We believe that this level of investigation will further broaden the clinical application of our clonal analysis methodology. Technological innovations will also advance the use of increasingly smaller inputs from sorted samples for deep genomic analyses. In particular new sequencing platforms offer the potential for low inputs which can remove the need for linear amplification of small purified samples (Zong et al., 2012). The development of more robust sequencing platforms and more efficient analysis pipelines will eliminate the use of array based studies. Thus we anticipate for each sorted population isolated from clinical samples of interest that a unified analysis of individual genomes, including somatic mutations, structural variations, and copy number aberrations will be derived from a single experiment. We believe that these analyses will provide an even deeper study of the clonal landscape of each tumor that can be advanced for improved patient care.

## Conflict of Interest Statement

The authors declare that the research was conducted in the absence of any commercial or financial relationships that could be construed as a potential conflict of interest.

## Footnotes

^1^http://www.bioinformatics.babraham.ac.uk/projects/fastqc/

^2^http://www.illumina.com/support/sequencing/sequencing_software/casava.ilmn

^3^http://picard.sourceforge.net
